# A multidisciplinary perspective on advancing genomic nursing in Portugal: roles, barriers and system-level solutions

**DOI:** 10.1007/s12687-026-00861-3

**Published:** 2026-02-17

**Authors:** Maria João Silva, Catarina Costa, Lídia Guimarães, Marcia Van Riper, Maria do Céu Barbieri Figueiredo, Milena Paneque

**Affiliations:** 1 ICBAS - School of Medicine and Biomedical Sciences of University of Porto, Rua de Jorge Viterbo Ferreira 228, 4050-313 Porto, Portugal; 2 Nursing School of Porto, University of Porto, Porto, Portugal; 3 i3S - Institute for Research and Innovation in Health, University of Porto, Porto, Portugal; 4 IBMC - Institute of Molecular and Cellular Biology, University of Porto, Porto, Portugal; 5 CGPP - Center for Predictive and Preventive Genetics, University of Porto, Porto, Portugal; 6 AAJUDE – Associação de Apoio à Juventude Deficiente, Porto, Portugal; 7 School of Nursing, University of North Carolina at Chapel Hill, Chapel Hill, NC USA

**Keywords:** Genomics, Interdisciplinary communication, Nursing, Nursing education, Patient care team, Health services accessibility

## Abstract

The rapid expansion of genomic science has reshaped healthcare delivery, creating new demands for interdisciplinary collaboration and highlighting the need to integrate genomics into nursing practice and education. This qualitative study explored how multidisciplinary genomic specialists, including medical geneticists, genetic counsellors and nurses, conceptualize nurses’ roles in genomic healthcare and the conditions required for effective integration in Portugal. Two online focus groups (*n* = 10) were conducted and the data were analyzed using Reflexive Thematic Analysis, following Braun and Clarke’s framework. Through an iterative and interpretive process, three themes capturing patterns of shared meaning were developed: the role of nurses in genomic teams, barriers to integrating genomics in nursing education and practice, and systemic and organizational solutions for genomic integration. Participants viewed nurses as pivotal to ensuring continuity, coordination and person- and family-centered care within genomic pathways. However, integration remains constrained by limited genomic literacy, fragmented educational provision, lack of regulatory recognition and insufficient institutional support. Participants highlighted the need for structured and longitudinal genomic education across undergraduate, postgraduate and continuing professional development levels, supported by clear role delineation, clinical supervision and professional recognition. They also emphasized advocacy and policy alignment as essential to building the infrastructure required for sustainable genomic nursing practice. Advancing nursing genomics in Portugal will require coordinated educational, organizational and regulatory reform, with nurses positioned as key contributors to equitable and person-centered genomic care.

**Supplementary Information:**

The online version contains supplementary material available at 10.1007/s12687-026-00861-3.

## Background

In more than two decades, since the completion of the Human Genome Project, rapid advances in genomic science and technology have profoundly transformed healthcare, reshaping diagnostic, preventive and therapeutic approaches (Zhu et al. 2024). Innovations such as next-generation sequencing, multi-omics integration and increasingly sophisticated bioinformatics now allow the interpretation of genetic variation with unprecedented speed and accuracy (Whitley et al. 2020). Concurrently, the integration of artificial intelligence and big-data analytics into genomic medicine has introduced new dimensions to this transformation (Dreisbach and Koleck 2020). Algorithms can now assist in risk assessment, variant interpretation and treatment recommendations, but these systems rely on professionals who can critically appraise their limitations, biases and ethical implications (Kurnat-Thoma 2020). Data literacy, understood as the ability to interpret and question machine-generated insights, has therefore become an essential component of safe and ethical genomic practice (Dreisbach and Koleck 2020).

At the same time, the rise of data-driven approaches has enabled the linkage of genomic, environmental and clinical information, supporting increasingly precise and personalized models of care (Hulick and Ilbawi 2024). These developments are redefining what it means to deliver evidence-based care, while creating new challenges for professionals who must balance innovation, ethics and human connection (Zhu et al. 2024). For nurses, this transformation has expanded both the scope and complexity of their roles, positioning them at the interface between rapidly evolving technologies and the lived experiences of patients (Calzone et al. 2024; Wang et al. 2023). Ensuring that nurses are adequately prepared to interpret genomic and data-driven information is therefore critical to maintaining person-centered, ethically grounded and equitable care.

In this changing landscape, nurses hold a central position in the clinical application of genomics, providing continuity of care and contributing to the integration of genomic information into everyday healthcare practice (Calzone et al. 2018b). They collect family histories, identify patterns of inherited risk, support consent processes, and facilitate informed understanding of genomic information to guide health-related decision-making in collaboration with patients and families (Dewell et al. 2024; Thomas et al. 2023; Tonkin et al. 2020b). Across these clinical interactions, nurses play a pivotal role in translating complex genomic data into meaningful, person-centered cate that bridges the gap between technology and lived experience (Keels et al. 2024).

Recent international assessments have consistently highlighted significant gaps in genomic literacy among healthcare professionals and the urgent need to strengthen genomics education across disciplines and health systems (Johnson et al. 2022; Parviainen et al. 2023; Wang et al. 2023). Despite the growing relevance of genomics to clinical care, preparation for genomics-informed nursing practice remains uneven across countries (Adejumo et al. 2021; Thomas et al. 2023). Although international frameworks have outlined core genomic knowledge and competencies for nurses (Calzone et al. 2024; Regan et al. 2019), recent evidence shows that their translation into education policy and clinical training remains limited (Himes et al. 2025b; Katapodi et al. 2023; Thomas et al. 2023). Consequently, many nurses continue to feel underprepared to deliver genomics-informed care, resulting in variable practice standards across healthcare settings (Ballad et al. 2024; Cao et al. 2025; Chow et al. 2023; Gusen et al. 2025; Hines-Dowell et al. 2024; Paneque et al. 2022; Schluter 2023).

While educational interventions have improved nurses’ conceptual understanding and confidence in genomics, evidence of broader or long-term curricular adoption remains limited (Clary-Muronda and Smith 2024; Smania et al. 2022; Yesilcinar et al. 2022; Zureigat et al. 2022). Variability in pedagogical design and evaluation approaches continues to limit comparability and the accumulation of evidence across settings (Joffe et al. 2024; Zureigat et al. 2022).

Emerging evidence also highlights the importance of connecting undergraduate, postgraduate and workplace learning, ensuring that genomic literacy evolves across the educational continuum and translates into clinical competence (Barbato et al. 2019; Chow et al. 2023; Kronk et al. 2024; Tonkin et al. 2020a).

Ongoing professional development and faculty training are therefore widely recognized as key components for advancing genomic education (Kronk et al. 2024; Mathis 2022; Smania et al. 2022). Initiatives such as online and postgraduate learning opportunities have been shown to enhance nurses’ conceptual understanding and confidence to teach or apply genomics in practice (Hines-Dowell et al. 2024; Kronk et al. 2023; Mathis 2022). Strengthening the pedagogical preparation of nurse educators is particularly important for embedding genomics across curricula and ensuring that educational reform translates into a genomically competent workforce (Himes et al. 2025a; Smania et al. 2022).

The integration of genomics into healthcare has reshaped clinical practice and expanded the range of professionals involved in genetic and genomic care. In this changing context, multidisciplinary collaboration has become a central feature of service delivery, involving physicians, genetic counsellors, laboratory scientists and nurses working together to provide coherent and person-centered care (Dewell et al. 2024; Espinoza-Moya et al. 2024; Ma et al. 2024).

Professional recognition and regulatory frameworks are increasingly viewed as key enablers of safe and sustainable genomic practice, shaping role definition, workforce planning and long-term service development (Carpenter-Clawson et al. 2023; Thomas et al. 2023).

In many healthcare systems, however, the participation of nurses in genomic practice remains limited and inconsistently defined (Giakoumidakis et al. 2025; Schluter 2023). The absence of formal recognition, role delineation and regulatory guidance continues to affect how nursing is positioned within genomic services (Limoges et al. 2024; Thomas et al. 2023). Unevenness in organizational structures and workforce capacity also contributes to differences in how genomics is implemented across clinical settings. Several authors have therefore called for structured frameworks that recognize genomic nursing as a distinct area of expertise, with defined competencies, certification pathways and opportunities for professional progression (Limoges et al. 2024; Thomas et al. 2023).

In Portugal, efforts to integrate genomics into nursing education and practice are still emerging. Previous analysis of national curricula revealed fragmented and predominantly biomedical coverage of genomic content, with limited attention to ethical, psychosocial and person-centered dimensions (Silva et al. 2025). At the same time, the national provision of genomic healthcare remains limited and uneven, reflecting workforce shortages and fragmented service structures that restrict equitable access and effective multidisciplinary collaboration (Costa et al. 2024a; Costa et al. 2024b; Paneque et al. 2022).

The scope and definition of nursing engagement in genomic settings remain limited, highlighting the importance of understanding how professionals working in these environments conceptualize nursing roles and the competencies needed for effective and sustainable practice.

## Aim

This study aims to identify the perspectives of multidisciplinary genomic specialists, including medical geneticists, genetic counsellors and nurses with experience in genomics, regarding the integration of genomics into nursing education and practice in Portugal. It seeks to describe perceived nursing roles within genomic healthcare, barriers and facilitators to integration, and the knowledge, skills and system-level conditions considered essential to support genomics-informed nursing practice.

## Methodology

### Study design and epistemological orientation

We conducted a qualitative, exploratory, and descriptive study using Reflexive Thematic Analysis (RTA) as outlined by Braun and Clarke (Braun and Clarke 2006, 2019, 2021). This approach shaped both analytic and methodological design, aligning with an interpretivist and reflexive paradigm that views meaning as co-constructed through interaction and researcher reflexivity rather than objectively discovered. The study aimed to understand how genomics is organized and practiced in Portuguese services, and what nurses need to participate effectively in that practice.

Online focus groups were used as an interpretive and dialogic method consistent with the epistemological stance of RTA, enabling participants to collectively construct and negotiate meanings through discussion and reflection. This design allowed geographically dispersed multidisciplinary genomic specialists to engage in interactive, reflective exchanges while reducing scheduling constraints. Secure screen sharing and brief anonymous prompts supported participation and helped attendees consider and prioritize ideas before speaking.

### Participants and recruitment pathways

Eligible participants were multidisciplinary genomic specialists working in genomics-related services in Portugal, including medical geneticists, genetic counsellors, and nurses whose routine practice incorporates genomic activities. Because the role of genetic nurse is not formally defined in Portugal, nurses were eligible when their day-to-day practice demonstrably involved genomic pathways or tasks. Inclusion criteria require current or recent involvement in delivering, coordinating or applying genomic care within the national health system. Recruitment proceeded in three complementary streams. First, calls for participation were disseminated via national bodies, namely the Portuguese Medical Association (College of the Medical Genetics Specialty), APPAcGen (Portuguese Association of Genetic Counselling Professionals), and SPGH (Portuguese Society of Human Genetics), with a request to circulate the invitation across their networks. Second, directors of all national medical genetics’ services were contacted and asked to distribute the invitation to their multidisciplinary teams. Third, contact-to-contact referrals were used to reach additional eligible multidisciplinary genomic specialists. All who expressed interest during the recruitment window and met the criteria were included. This recruitment process yielded two focus groups with five participants each, totaling ten multidisciplinary genomic specialists.

The authors of this study are six women from diverse disciplinary, professional, and cultural backgrounds, including nursing, psychology, and genetic counselling, with experience across clinical practice, education, and research. The team represents Portuguese, Cuban, and American perspectives, which enriched the interpretive process and supported critical reflexivity. Their distinct disciplinary identities, professional trajectories, and cultural contexts shaped how the topic was approached and interpreted. This diversity is consistent with the reflexive nature of RTA, which acknowledges the researcher’s active role in meaning-making and interpretation.

### Dataset generation

Data ware generated between May and June 2025 during private meetings on the Zoom platform using an institutional account protected by a password. Session 1 lasted 1 h 33 min and included three medical geneticists and two nurses who applied genomics in their practice. Session 2 lasted 1 h 17 min and included one nurse, two genetic counsellors, and two medical geneticists. A semi-structured guide, prepared a priori from the study aims and relevant literature, circulated in advance with a concise session plan (Appendix A). The guide invited discussions of service organization and workflows, professional roles and interfaces, competencies required for safe and effective genomic care, gaps and training needs for nurses, contextual enablers and barriers, and practice-oriented recommendations.

The first author facilitated the conversations in a manner consistent with the epistemological stance of RTA, fostering dialogic engagement and collective meaning-making rather than neutral moderation. A second researcher experienced in qualitative inquiry supported the sessions and maintained reflexive notes documenting contextual observations and analytic insights throughout. To encourage broad participation in the online setting and to support reflective deliberation, Mentimeter, an interactive presentation tool that enables real-time anonymous audience input, was used at a small number of pre-specified moments to elicit contributions from all attendees; these responses were displayed to the group in real time, exported in aggregate, and treated as reflective prompts that complemented, rather than replaced, the dialogic data generated through interaction. Sessions were audio-recorded in Zoom, and automated transcripts were reviewed and refined to ensure they captured the dialogic and contextual features of the discussions, producing meaning-oriented textual accounts suitable for reflexive analysis.

### Data analysis

The focus group transcripts were analyzed using RTA as described by Braun and Clarke (Braun and Clarke 2006, 2019, 2021, 2024). RTA was selected for its methodological rigor and suitability for exploring complex, context-dependent professional experiences in an interpretive and reflexive manner. This approach enabled the systematic identification and interpretation of patterns of shared meaning within the data, providing insights into perceptions and practices related to genomics across professional roles in Portugal.

The analytic process was informed by the diverse disciplinary and cultural perspectives of the research team, as described above.

The analytic process followed Braun and Clarke’s six-step framework (2006). Initial readings and coding were undertaken by two researchers who independently identified key features of the data relevant to the research aims. These preliminary codes and conceptual diagrams were then reviewed and discussed with a third researcher to explore interpretive connections and ensure analytic depth. Codes were examined for conceptual and contextual relationships and grouped into preliminary themes, which were refined through iterative team discussions. All themes were subsequently revisited, reflected upon, and debated by the wider author team to ensure coherence, richness, and alignment with the interpretive focus of the study. Themes were further refined and clearly labelled to reflect their central organizing ideas, and a thematic map was developed to illustrate the relationships between themes and subthemes. Although some ideas were mentioned less frequently, these were retained when they offered conceptual relevance or illuminated important aspects of the phenomenon. Consistent with Reflexive Thematic Analysis, themes were conceptualized as patterns of shared meaning underpinned by a central organizing concept rather than by frequency.

The study was reported in accordance with the Reflexive Thematic Analysis Reporting Guidelines (RTARG; Braun and Clarke 2024), which emphasize methodological coherence, reflexive openness, and transparency. A completed RTARG checklist is provided as Appendix B.

### Ethical considerations

The study received approval from the Committee for Ethical and Responsible Conduct of Research (CECRI, i3S; Ref. 5/CECRI/2025). Participants received an information sheet and a written consent form in advance, and verbal informed consent, including consent to record, was obtained on Zoom before recording commenced. Audio files were stored on password-protected institutional drives with restricted access and were deleted after manual verification of the transcripts. Pseudonymized transcripts were retained for analysis in accordance with the General Data Protection Regulation.

## Analytic narrative

### Participants’ profile

Participants were multidisciplinary genomic specialists working in diverse clinical and institutional contexts across Portugal. Their professional backgrounds encompassed medical genetics, nursing, and genetic counselling, reflecting the collaborative nature of genomic healthcare. All were directly involved in genomic activities, whether in clinical practice, education or research, and brought experiential knowledge shaped by their everyday work. Rather than aiming for representativeness, the composition of the groups was intended to elicit a breadth of perspectives and to promote dialogic exchange among professionals with complementary expertise. This diversity enriched the interpretive process, as participants’ narratives were grounded in their lived engagement with genomic care and offered insight into both shared understandings and the contextual nuances that shape genomic practice in Portugal. An overview of participants’ professional profiles is provided in Table 1. In this study, the category ‘genetic counsellor/nurse’ refers to registered nurses who hold formal postgraduate training in genetic counselling (MSc) and practice in this specialized capacity. The category ‘nurse’ refers to registered nurses working in genomic healthcare settings without specialized training in genetic counselling; because the designation ‘genetic nurse’ is not formally recognized in Portugal, these participants are classified according to their nursing background and current genomic practice.

**Table 1:** Participant profile of multidisciplinary genomic specialists

Participant	Gender	Year of professional experience	Professional background	Institution (region)
P1	F	> 20 years	Genetic counsellor / Psychologist	North
P2	M	5–10 years	Medical geneticist	North
P3	M	10–20 years	Medical geneticist	North
P4	M	5–10 years	Medical geneticist	Lisbon
P5	F	10–20 years	Genetic counsellor /Nurse*	North
P6	F	> 20 years	Medical geneticist	North
P7	F	> 20 years	Nurse**	North
P8	F	> 20 years	Nurse**	Lisbon
P9	F	> 20 years	Genetic counsellor/ Nurse*	Autonomous Region of the Azores
P10	F	5–10 years	Medical geneticist	Lisbon

*Genetic counsellor/nurses: registered nurses who completed postgraduate training in genetic counselling (MSc).

** Nurses: registered nurses working in genomic healthcare settings without formal postgraduate training in genetic counselling.

This description aligns with the Reflexive Thematic Analysis Reporting Guidelines (Braun and Clarke 2024), privileging contextual transparency over demographic detail.

### Genomics integration in nursing education and practice

**Fig. 1:**
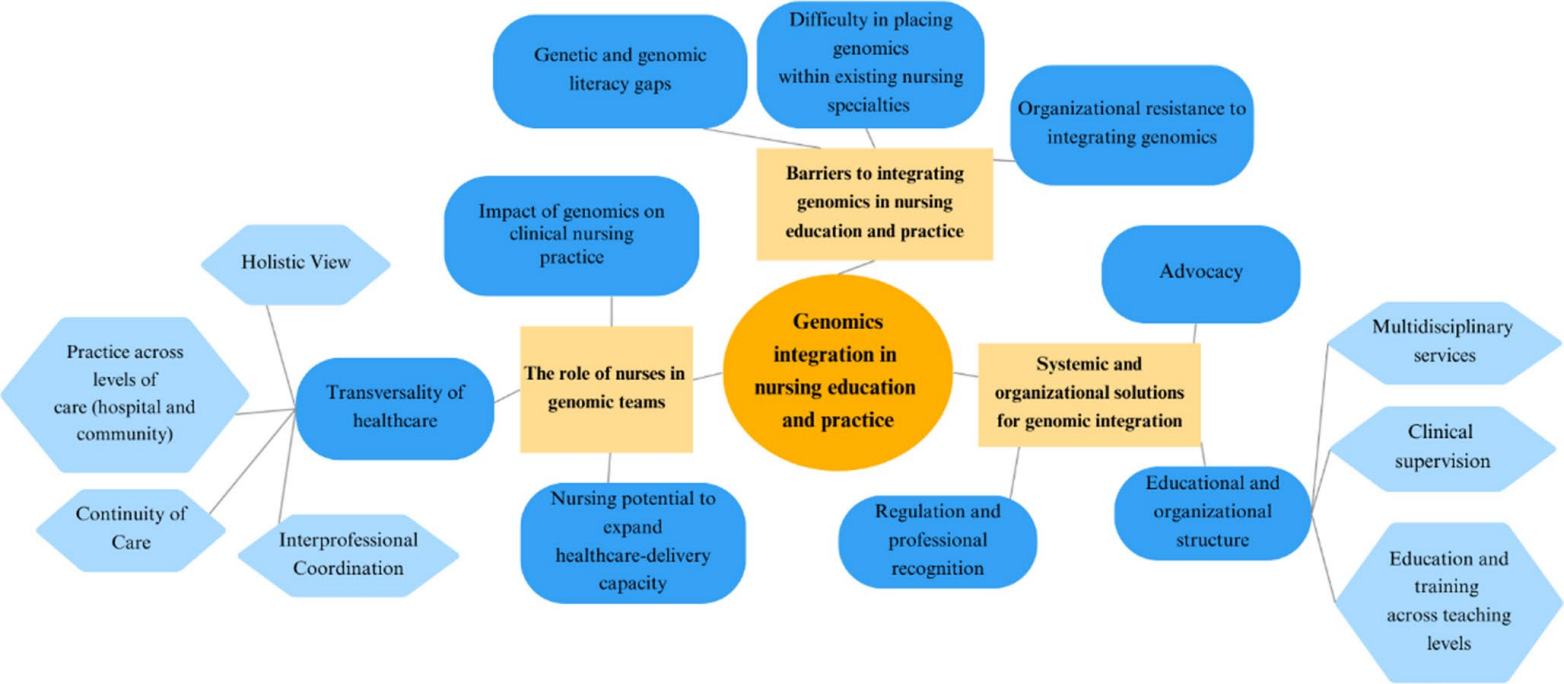
Themes and their conceptual relationships

Through reflexive thematic analysis, three overarching themes were developed to represent shared meanings in participants’ accounts regarding nurses’ contributions to genomic healthcare. Together, these themes illustrate how multidisciplinary genomic specialists conceptualized the current and potential contribution of nurses, as well as the conditions that enable or constrain integration in education and practice. These themes were: (1) the role of nurses in genomic teams, (2) barriers to integrating genomics in nursing education and practice, and (3) systemic and organizational solutions for genomic integration. Illustrative quotations are presented throughout to amplify participants’ voices and support interpretation. The thematic structure developed through the analysis is summarized in Fig. 1, which visually represents the three main themes and their conceptual relationships.

### Theme 1 — the role of nurses in genomic teams

This theme captures how multidisciplinary genomic specialists conceptualized the place of nurses within genomic healthcare and how their existing roles were perceived to align with the evolving demands of genomic medicine. Participants described nursing as central to the organization and continuity of care, with responsibilities extending from clinical assessment and patient support to coordination across services and specialties. Genomics was understood as deepening and expanding these functions rather than redefining them, reinforcing the profession’s integrative and person-centered orientation. The following subthemes illustrate this contribution across three interrelated dimensions: the growing impact of genomics on clinical nursing practice, the potential of nurses to expand healthcare-delivery capacity, and the transversal nature of nursing roles across care settings and disciplines.

### Impact of genomics on clinical nursing practice

Multidisciplinary genomic specialists recognized genomics as increasingly integral to nursing practice. They situated its relevance at the point of care, where genomic information guides clinical judgement, communication and follow up, and at the organizational level, where nursing preparedness shapes access and continuity of genomic care. They emphasized forms of incorporation that were consistent with nursing’s person and family centered philosophy, enabling meaningful contribution without reducing the role to a narrow technical function.

Participants portrayed genomics as increasingly essential to timely, accurate care, linking next-generation sequencing to higher diagnostic yield and earlier case resolution. They highlighted that genomic information now extends beyond heredity and risk estimation to inform therapeutic eligibility, stratification and personalized care planning. In this view, the value of genomics lies not only in detecting variants but in integrating results into assessment, shared decision-making, communication with patients and families, and longitudinal follow-up, domains in which nursing plays a continuous role.

*“Given the current clinical relevance of genomic data—far beyond heredity*,* risk and prevention—it is clear that nurses need this knowledge… It is not only about diagnosing or identifying genetic variants. Genetic results need to be integrated into clinical assessment*,* explained and discussed with patients and families*,* and followed over time*,* as they influence decisions and care beyond the moment of testing.” (P7)*.

*“The diagnostic yield and sensitivity have improved with Next-Generation Sequencing (NGS). Today*,* we also assess therapeutic eligibility; however*,* the focus extends beyond diagnosis or test results—nurses play a complementary role in the diagnostic process and remain involved throughout the continuum of care.” (P4)*.

### Nursing potential to expand healthcare-delivery capacity

Multidisciplinary genomic specialists argued that preparing nurses in genomics could increase care-delivery capacity by addressing the shortage of medical geneticists/counsellors and the resulting long waiting lists. The surge in genetic testing has intensified demand for communicating results and supporting patients, while consultations are prolonged by tasks that trained nursing teams could perform, thereby shortening medical consultations and enabling more timely access. Nurses’ contributions were framed as complementary to medical roles; participants cautioned against a purely economistic view of using nurses only to accelerate throughput. They also noted that work around variants of uncertain significance (VUS) could be appropriately guided by non-medical professionals with suitable preparation.

*“There is a severe shortage of medical geneticists to meet the current demand in Portugal. Waiting lists are long*,* consultations are time-consuming*,* and many of the tasks that prolong visits could be undertaken by a nursing team trained for this purpose.” (P3)*.

*“The role of nurses is often viewed through the lens of reducing medical consultation time and thereby increasing the number of consultations*,* which makes sense*,* but reflects a more economistic perspective. Nursing contributions can extend beyond this view*,* encompassing roles in communication*,* decision-making support*,* and continuity of care” (P6)*.

*“Another very current issue is variants of uncertain significance*,* which could also be managed by non-medical professionals.” (P4)*.

### Transversality of healthcare

This subtheme captures how participants described the transversal nature of nursing within genomic care pathways, reflecting how nurses’ roles extend across clinical settings, levels of care and professional boundaries. Participants described this transversality as grounded in the continuity and relational depth of nursing work, which enables coordination of care, communication across disciplines, and sustained engagement with individuals and families over time. Four interrelated dimensions illustrate this integrative function: continuity of care, a holistic view of practice, interprofessional coordination, and articulation across hospital and community settings.

#### Continuity of care

Multidisciplinary genomic specialists described nurses as central members of healthcare teams and services. Their continuous presence across settings and across the lifespan, proximity to patients and families, and transversal contribution to care enable the coordination of communication and transitions of care. In practice, nurses already carry out navigation across pathways of care, information exchange and continuity work and specific preparation in genomics would strengthen these functions. Participants noted that nurses’ place within multidisciplinary teams facilitates the operationalization of genomic processes in clinical contexts, from preparing and supporting patients to aligning inputs across professionals and specialties. This existing centrality also supports recognition of family implications of genetic findings and sustained person and family centered engagement over time.

*“Everything has implications for individuals and families*,* but also for teams; multidisciplinary collaboration and organization among members will be key. The nurse has an important role here.” (P7)*.

*“Having nurses in genetics services and multidisciplinary teams adds value; genetics often has family implications*,* and nurses can get closer to patients than doctors.” (P4)*.

Multidisciplinary genomic specialists also portrayed nurses as case managers who coordinate the person’s and family’s journey through genetic care. Core responsibilities include eliciting clinical and family history with pedigree construction, preparing for testing through information and consent support, explaining results in accessible language, and identifying and managing emotional responses including distress, with referral to psychology when indicated. Nurses provide decision support, maintain open channels of communication after results, and organize structured follow up, including telephone and teleconsultation contacts. They also triage and prioritize referrals in line with therapeutic or surgical decisions, identify healthy relatives at risk, and coordinate their timely access to genetics clinics, which sustains continuity across services. This role was framed as person and family centered, translating technical findings into understandable information and practical next steps while maintaining longitudinal engagement.

*“We triage incoming consultation requests and prioritize them in line with therapeutic and surgical decisions. We also manage the family dimension of care*,* identifying hereditary patterns and coordinating genetic consultations for healthy relatives at risk.” (P5)*.

*“Many times*,* the patient and the family cannot understand or decode medical language. And sometimes*,* at that moment*,* they do not feel comfortable to ask questions*,* and we must be informed and have this knowledge to correctly inform that family.” (P5)*.

*“Nurses know how to engage with the patient’s care pathway*,* mobilize resources*,* and define or prioritize genetic diagnosis to enable timely therapeutic decisions.” (P7).*

*“It is important to have an easier*,* more open channel of contact with a nurse who can make a call a few days later*,* or check how the person is adapting to the information*,* whether they have integrated it or understood it*,* whether they have questions*,* whether they have shared it with the family…” (P6)*.

#### Holistic view

Multidisciplinary genomic specialists highlighted features of nursing education and practice that facilitate the integration of genomics. Foundational preparation in biomedical subjects such as physiology, anatomy and microbiology provides a base for acquiring genomic concepts. A holistic orientation, strong communication skills and a person- and family-centered approach position nurses to translate technical results into meaningful support, decision making and follow up. Participants viewed nurses as well placed to upskill quickly, arguing that existing knowledge, competencies and attitudes can be leveraged to deliver genomics-informed care across settings and across the lifespan. This facilitative profile is both relational and practical, combining proximity to patients and families with the ability to coordinate inputs across disciplines and to sustain engagement over time.

*“In my opinion*,* nurses have excellent preparation to work in genetics and genomics. If we consider knowledge*,* skills and attitudes*,* the attitude*,* so central in nursing education*,* should be highlighted as a major facilitator. Nurses have an ideal profile*,* with training centered on the patient*,* the family and the continuum of care; they are indispensable to genetic care.” (P1)*.

*“From my experience*,* nurses are clearly very well positioned for training. They quickly become competent*,* have the necessary foundations*,* and can then help implement more genomic*,* more personalized medicine.” (P2)*.

#### Interprofessional coordination

Multidisciplinary genomic specialists emphasized the nurse as intermediary between the patient and the wider clinical team. This role centers on making patient values, questions and concerns visible in clinical discussions and facilitating the flow of information across specialties so that messages are consistent and plans are coherent. Nurses translate technical language, reconcile differing professional perspectives and help the team reach shared understandings. Active participation in multidisciplinary meetings was seen as essential, where nurses broker communication between psychology, surgery, radiology and genetics, clarify roles and next steps, and ensure that agreed actions are communicated back to patients and families. Preparation in genomics was understood to strengthen the credibility and effectiveness of this liaison work.

*“Nurses are often the patients’ representative and the link across the team; it is crucial they have training in this area.” (P7)*.

*“In multidisciplinary meetings we bridge with psychology*,* surgery and radiology… and interpret genetic study results with the medical geneticist.” (P9)*.

#### Practice across levels of care (hospital and community)

Multidisciplinary genomic specialists described a versatile nursing role that operates across hospital and community settings. Proximity to people and continuity of care position nurses to identify genetic needs early, signal families at risk, provide first line information and maintain follow up outside specialized clinics. The role extends prevention efforts and widens access beyond tertiary centers, reducing geographic and organizational inequities. Participants called for coordinated pathways linking primary, secondary and tertiary care and for inter hospital collaboration so that referrals, counselling and surveillance are coherent across sites. They highlighted the value of a national approach and the creation of liaison roles within Local Health Units to connect specialized genetics with primary care and community teams. In this view, embedding genomics across levels of care was understood by participants as promoting earlier recognition of risk, timely escalation to genetics units and sustained support within routine community practice.

*“This has to move beyond the large hospital centers*,* and these reference professionals will also be key in inter-hospital care and in primary care. We need a national strategy for these individuals that involves all care networks: primary*,* secondary and tertiary.” (P7)*.

### Theme 2 - barriers to integrating genomics in nursing education and practice

This theme explores how participants understood the structural, organizational and educational barriers that hinder the integration of genomics into nursing education and practice. Participants described challenges that operate at multiple levels, from limited genomic literacy among professionals and decision-makers to institutional resistance and regulatory gaps that constrain role development. They also highlighted difficulties in aligning genomics with existing nursing specialties, resulting in fragmented curricular inclusion and uneven implementation across clinical contexts. Together, these accounts reveal systemic and organizational conditions that continue to limit the coherent integration of genomics within nursing.

### Genetic and genomic literacy gaps

Participants described literacy gaps not only among health professionals but across the wider ecosystem, including the public, policy makers and administrators. They perceived that low system-level literacy weakened prioritization and administrative support for education and service development. Within clinical settings, many nurses reported little or no formal genetics in undergraduate education and only developed knowledge after joining multidisciplinary teams, while limited literacy was also observed among non-genetics medical specialties. These patterns were seen as constraining the consistent application of key concepts in practice, including inheritance patterns, family history and pedigree taking, interpretation of variants (notably variants of uncertain significance), and pre and post-test counselling with attention to psychosocial impact. Overall, undergraduate provision in genetics remains limited and misaligned with current genomic advances, leaving a foundational gap that hinders safe, person and family centered care in genomic contexts.

*“There is a broader dimension: low literacy in society*,* among policymakers and administrators.” (P1)*.

*“When I joined the multidisciplinary team*,* the training was given to me within the team*,* by the doctors in the team*,* because my foundational training was practically non-existent.” (P8)*.

*“Genetic literacy is also an issue within the medical profession; physicians in other specialties have limited training in genetics. Undergraduate education offers little genetics and remains limited in relation to advances in genomics.” (P2)*.

### Organizational resistance to integrating genomics into nursing practice

Participants described institutional distance from genomic care needs, combining limited awareness among leaders with fragile human resource arrangements. Decision makers and professional bodies were portrayed as insufficiently informed about educational and operational requirements, which translated into unclear policies, reluctance to invest in training and slow or absent administrative responses. Participants also noted that the regulatory board has not yet formally recognized genetic counselling as an additional nursing competence, which they perceived as reflecting limited institutional prioritization of nursing roles in genomics. At the operational level, the absence of designated nursing posts in genetics and frequent redeployment to other units disrupted continuity, prevented specialization and diluted expertise. Credible integration was said to require explicit, preferably full time, nursing positions within genetics units, backed by administrative mandates and resources, alongside a shift in leadership mindsets to reflect contemporary genomic practice rather than traditional task views.

*“There is goodwill in some situations*,* but there is no administrative response from policymakers.” (P1)*.

*“When we speak with decision-makers*,* even after many years of working in this area*,* they often do not grasp the concept because they remain anchored in outdated*,* task-based views of care.” (P7)*.

*“It is essential*,* to unlock and optimize integration*,* that nursing posts be established within genetics services*,* or at least that nurses are formally allocated to genetics units.” (P6)*.

### Difficulty in placing genomics within existing nursing specialties

Participants characterized genomics as transversal to multiple areas of nursing practice, including maternal and child health, community, pediatrics and oncology. Because the Portuguese nursing specialty framework is structured into distinct domains with defined scopes and competencies, new content is usually assigned to a single domain. Genomics does not align with any one domain, which leaves it without a defined curricular placement or practice designation, contributing to marginal status in both education and service delivery.

Although the complexity and specificity of genomics warrant postgraduate development, the present model offers limited pathways for structured uptake. Participants pointed to two feasible routes: formal recognition of a cross-specialty competence that sits outside existing domains, or creation of a distinct pathway in genomic nursing. Without such options, integration is likely to remain fragmentary and reliant on local initiative, leading to inconsistent curricular coverage, uneven preparedness of graduates, fragmented role allocation in clinical settings and difficulties in planning supervision, assessment and career progression. A clear regulatory settlement and workforce design were identified as prerequisites for coherent adoption across programs and services.

*“There is a conception of nursing structured around specialties*,* so anything new has to be placed within one. Genetics does not fit any single specialty; it spans them all and*,* on its own*,* cannot be integrated under the current conception.” (P7)*.

*“Unless there is a vision to create a cross-cutting competence outside the existing nursing specialty tracks*,* meaningful integration will not be possible.” (P5)*.

*“It is an essential area*,* but it continues to lack a defined place in both education and clinical practice. As a result*,* preparation varies widely between programs*,* roles are allocated inconsistently across services*,* and it becomes difficult to plan supervision*,* assessment*,* and career progression in a coherent way.” (P1)*.

### Theme 3 — Systemic and organizational solutions for genomic integration

This theme focuses on the strategies proposed by participants to enable the effective and sustainable integration of genomics within nursing education and practice. Multidisciplinary genomic specialists emphasized the need for a coherent system that aligns educational preparation, professional regulation, team organization and policy support. Their accounts highlighted interconnected priorities, including the development of structured educational pathways, clear interprofessional role delineation, formal clinical supervision, professional recognition and active advocacy. Together, these strategies reflect a collective vision for building the infrastructure and culture required to embed genomics across nursing and healthcare systems.

### Educational and organizational structure

Participants viewed the effective integration of genomics in nursing as reliant on a coherent infrastructure that connects educational preparation, team organization, and professional support mechanisms. They emphasized that sustained genomic practice requires aligned investment in education, well-defined multidisciplinary collaboration, and formal structures of clinical supervision to ensure quality, safety, and emotional support for professionals.

#### Education and training across teaching levels

Participants called for a coherent approach to education that begins in undergraduate programs and continues through postgraduate and in-service training. At entry level, all nurses should attain minimum genetic and genomic literacy, with content introduced early and developed longitudinally across the degree from simple to more complex concepts. Provision should be transversal rather than confined to a single year, so that concepts are revisited and applied in different clinical contexts. This baseline was viewed as essential even for those who will not specialize, both to improve day-to-day communication with patients and families and to stimulate interest in joining genetics teams.

Core knowledge identified for undergraduate programs included: basic principles from DNA to phenotype; Mendelian and non-Mendelian inheritance, penetrance and expressivity; the distinction between constitutional and somatic genetics; family history and pedigree taking with recognition of red flags and referral criteria; genomic testing indications, pathways, limitations and performance; interpretation and communication of results; introductory principles of variant interpretation; ethical and legal issues such as consent, privacy, data sharing and discrimination; psychosocial impact of genetic risk and diagnosis, including recognition of distress and routes to support; communication competencies for person and family centered practice, including use of family assessment tools.

For the existing workforce, participants advocated structured, practice-based continuing education tailored to clinical settings, using interactive methods, supervised application and case discussions. Given the small pool of trainers across many nursing programs, they recommended drawing on the expertise of nurses and teams already working in genetics to teach and mentor others, thereby translating “know-how from practice” into education. Deeper, practice-oriented learning should be available at postgraduate level; however, participants noted few formal routes and the absence of master’s-level preparation for genetic nurses in Europe, despite the role being recognized and valued in care. Overall, education should be intentional, aligned with service needs and designed to produce observable gains in practice.

*“We now truly need to push for genetics to be included in basic training*,* because clinical need will then draw nurses into multidisciplinary teams.” (P7)*.

*“It is important that this training is included from undergraduate education*,* with subsequent development at postgraduate level—perhaps not a formal specialty*,* but at least a postgraduate program.” (P8)*.

*“There is currently no master’s-level pathway in Europe for training genetic nurses*,* yet it remains a recognized profession with a unique*,* crucial role in genetic healthcare” (P1)*.

#### Multidisciplinary services

Participants stressed the need for explicit interprofessional role delineation in genomic care to prevent overlaps and gaps, support professional autonomy and improve team efficiency. They advocated developing a competence map that makes clear what can be shared across professions and what must remain restricted, with agreed escalation and supervision routes. Overlap was not seen as problematic if boundaries, responsibilities and lines of authority are transparent and respected in multidisciplinary meetings. Clear scopes would also enable coherent handovers between genetics, surgery, radiology, psychology and nursing.

*“We should not be afraid of some overlap; there are tasks I can do as a physician that a nurse can also do.” (P3).*

*“Ultimately, a multidisciplinary team works when roles are well defined and understood.” (P2).*

#### Clinical supervision

Alongside delineation, participants called for formal clinical supervision for nurses working in genetics to safeguard quality and mitigate burnout. Work with families facing complex decisions and high emotional load requires structured support: reflective debriefing, case discussion with senior clinicians, and training in emotional self-management embedded in nursing curricula. Supervision was framed as a core component of safe genomic practice rather than an optional add on.

*“It is important that nurses have tools to manage their own emotions and the impact they are having on the person in front of them; such self-management competencies are also highly pertinent in nursing curricula.” (P6).*

### Regulation and professional recognition

Participants proposed formal recognition of genomic competencies for nurses through a professional title or certification. Such regulation was seen as essential to legitimize differentiated clinical practice, clarify roles within teams and create coherent career pathways. Alignment with recognized professional domains and competency frameworks would support motivation and ongoing updating, both as a general competence for all nurses and as a route for vertical progression into advanced practice for some. Looking ahead, several participants envisioned a dedicated organizational structure for genomic nursing, with delineated areas of expertise such as nephrogenetics, neurogenetics, cardiogenetics and metabolic genetics. They also pointed to the influence of European reference networks and to the value of national, European and international recommendations in shaping policy, staffing expectations and educational requirements. In this view, regulatory recognition operates as a lever for service design, workforce planning and quality assurance, ensuring that preparation and scope of practice are consistently defined across settings.

*“Alignment with recognized areas and competencies would foster motivation and updating*,* not only as a general competence but also as vertical progression into specialization.” (P1)*.

*“In the longer term this will be a separate college*,* a new specialty with multiple areas of competence*,* from nephrogenetics to neurogenetics and cardiogenetics.” (P7)*.

*“European networks help by effectively requiring that hospitals include a member of this professional group; administrations then place a nurse in post. Recommendations at national and international level also help regulate roles and training.” (P7)*.

### Advocacy

Participants emphasized advocacy as a core mechanism to integrate genomics in nursing education and practice. Nurses and their professional and regulatory bodies were seen as key actors to raise literacy, articulate the distinctive nursing contribution, and secure policy and organizational support. Proposed actions included targeted briefings with the regulatory board and curriculum committees, development of position statements and competency specifications, and structured engagement with hospital administrators to align staffing, roles and training. Advocacy was framed as multi-level: nationally, to influence standards and recommendations; institutionally, to shape curricula and continuing education; and locally, to support team level implementation and clarify scopes of practice. The intended outcomes were consistent inclusion of genomics in undergraduate and postgraduate provision, investment in educator capacity, and recognition of designated nursing roles in genetics services.

*“It is important to keep raising awareness in postgraduate and specialty programs; advocacy with the regulatory board is needed to set out what integrating genomics into nursing entails*,* where we can make a difference*,* and what we add.” (P5)*.

## Discussion

This analysis highlights that multidisciplinary genomic specialists view nurses as important actors in bridging genomic science and everyday clinical care, yet significant educational, organizational and regulatory challenges continue to limit their contribution. These interpretations resonate with international evidence suggesting that, although genomics is increasingly embedded in healthcare systems, the translation of genomic knowledge into nursing practice remains fragmented and inconsistent across countries (Calzone et al. 2018a; Himes et al. 2025a; Thomas et al. 2023; Walker et al. 2024; Zureigat et al. 2022). Interpreting these insights within a broader perspective suggests that advancing nursing genomics is understood to require action at multiple levels, education, professional recognition and health system organization, supported by a culture that values the relational and ethical dimensions of care alongside technological innovation.

Across contexts, nurses are recognized as the connective tissue of genomic care, linking laboratory processes with the relational, communicative and ethical dimensions of patient support (Dewell et al. 2024; Tonkin et al. 2020a; Tonkin et al. 2020b). Multidisciplinary genomic specialists in this study described nurses as coordinators of care and advocates for families navigating genomic information, ensuring coherence across disciplines and continuity across services. These interpretations align with international evidence and provide additional insights suggesting that when nurses are formally integrated into genomic teams, they facilitate communication, enhance continuity and improve patient engagement (Cao et al. 2025; Costa et al. 2024; Kawasaki et al. 2021; Ma et al. 2024; Schluter 2023; Tonkin et al. 2020a). In these settings, nurses contribute beyond technical tasks, assuming interpretive, ethical and psychosocial functions that sustain person-centered care in data-intensive environments. For Portugal, where genomic healthcare provision remains fragmented, recognizing and formalizing these integrative nursing roles was viewed as a means to support more coherent, equitable and sustainable genomic service delivery (Costa et al. 2024a; Costa et al. 2023).

Educational preparation was understood as both the foundation and the key challenge for genomic integration. Participants underscored the persistence of curricular gaps and limited opportunities for professional development, noting that most nurses acquire genomic literacy informally, through experience rather than structured training. Similar challenges have been reported globally, where rapid advances in genomics have outpaced educational reform and left faculty underprepared (McLaughlin et al. 2024; Nisselle et al. 2024; Smania et al. 2022; Thomas et al. 2023). In contrast, countries that have achieved meaningful progress, such as the United States and the United Kingdom, implemented systemic, multi-level strategies (Calzone et al. 2024; Carpenter-Clawson et al. 2023; Connors et al. 2025; Cowley et al. 2025; Tonkin et al. 2020b). These experiences illustrate that sustainable progress depends on longitudinal curriculum integration, investment in educator preparation and alignment between education, clinical service and professional regulation. To further contextualize these interpretations, international evidence points to additional systemic factors that shape the feasibility of integrating genomics into nursing education. One recurring challenge concerns academic and clinical faculty capacity, as many educators report limited preparation and insufficient institutional support to teach genomic content confidently (Himes et al. 2025b; Himes et al. 2025a; Mathis 2022). Access to clinical learning opportunities is also constrained, particularly in health systems where genomic services are concentrated in specialized centers, which reduces students’ exposure to genomic practice. When direct clinical experience is not feasible, alternative pedagogical approaches such as simulation, standardized patients or case-based learning can provide structured opportunities to apply genomic concepts (Dwyer et al. 2025; Zureigat et al. 2022). Participants also highlight the value of academic–practice partnerships, showing that collaboration between clinicians and educators strengthens the alignment of educational content with evolving clinical realities and supports the sustained development of genomic competence within the workforce. Ensuring the continuity of these efforts requires long-term institutional planning, since turnover among academic and clinical staff can challenge the continuity of organizational knowledge and disrupt the stability of genomic education (Himes et al. 2025a; Smania et al. 2022).

Although this study reflects the Portuguese context, the issues identified are not unique to Portugal. Many health systems with emerging genomic infrastructures face similar challenges in ensuring consistent educational preparation, faculty competence, and regulatory alignment (Ballad et al. 2024; Chow et al. 2023; Giakoumidakis et al. 2025; Ramírez-Baraldes et al. 2024; Yesilcinar et al. 2022). This analysis therefore offers transferable insights for other contexts seeking to strengthen nursing capacity for genomics-informed care through coherent, system-level strategies.

However, multidisciplinary experts in this study emphasized that education alone may not sustain genomic nursing practice if structural and regulatory conditions remain unchanged. Participants in this study highlighted the absence of designated nursing posts in genetics services, ineffective institutional prioritization and the lack of professional recognition as barriers to integration. These interpretations resonate with international analyses identifying regulation and role delineation as key enablers of safe and effective genomic practice (Calzone et al. 2024; Tully et al. 2020). Defined core knowledge, establish competencies, supervision structures and accreditation pathways were viewed as mechanisms that legitimized practice, improved quality assurance and fostered professional identity (Cao et al. 2025; Carpenter-Clawson et al. 2023; Guimaraes et al. 2024; Gusen et al. 2025; Himes et al. 2025a; Kronk et al. 2024; Regan et al. 2019). Regulation thus operates not only as an administrative mechanism but as a transformative force that shapes workforce design, reinforces accountability and enables the redistribution of genomic responsibilities across teams.

From the Portuguese perspective, this aligns with ongoing debates about the importance of professional recognition. The introduction of an officially recognized genomic nursing competence was perceived as a potential catalyst for integration, clarifying scopes of practice, legitimizing advanced roles and supporting career progression. Beyond symbolic validation, such recognition would enhance service capacity by enabling nurses to assume defined roles in communication, consent, and follow-up, thereby complementing the work of genetic counsellors and physicians. In systems under pressure from workforce shortages, as Portugal currently experiences, expanding nursing involvement through regulated roles could shorten waiting lists, strengthen continuity of care and foster multidisciplinary efficiency. This form of structural empowerment echoes the principle of transforming nursing practice articulated in international frameworks, in which regulation and education operate together to elevate nursing’s contribution to precision health.

Taken together, these interpretations reaffirm that integrating genomics in nursing requires a dual transformation: building educational and regulatory infrastructures while fostering a professional culture that values nursing’s interpretive, relational and ethical roles. When adequately supported, nurses were described as not only implementers but co-architects of genomic healthcare, helping translate scientific discovery into equitable, person-centered practice that advances both health outcomes and professional development.

## Strengths and limitations

This study offers a situated and in-depth interpretation of how multidisciplinary genomic specialists in Portugal conceptualize the role of nurses within genomic healthcare and identify conditions for integrating genomics into nursing practice and education. A major strength lies in the inclusion of diverse professional perspectives, medical geneticists, genetic counsellors, and nurses, which enabled a comprehensive view of team dynamics, competencies, and system-level barriers. The use of RTA, guided by Braun and Clarke’s framework, supported a nuanced, interpretive engagement with participants’ accounts. Reflexive attention to the research process and transparency in analytic decision-making strengthened the coherence and trustworthiness of this interpretation.

As with all qualitative, interpretive work, these insights are context-bound rather than generalizable. The analysis reflects the perspectives of professionals working in Portuguese genomic services, and different meanings might emerge in other healthcare systems or regulatory contexts. Accordingly, the findings are best understood in terms of their transferability, offering analytic insights that may inform contexts with similar levels of genomic integration rather than direct generalization. Although the inclusion of multiple disciplines deepened the interpretative scope, the dataset was deliberately focused rather than extensive, privileging analytic depth over breadth. The study did not include nurses without experience in genomic contexts or health policy decision-makers, which limits the ability to capture broader system-level or frontline nursing perspectives. We also acknowledge that participants’ professional commitment to genomics may have shaped how they articulated opportunities for nursing integration; likewise, our own positionality as researchers with backgrounds in nursing, psychology, and genetic counselling inevitably informed how we engaged with the data and constructed meaning. This interpretive lens, while enhancing analytic sensitivity, also represents a limitation inherent to reflexive qualitative research. Future studies could extend these interpretations by engaging broader nursing populations and health policy stakeholders to further explore systemic perspectives on genomic integration.

## Final considerations

This study offers a reflexive interpretation of how multidisciplinary genomic specialists conceptualize the role of nurses within genomic healthcare and the conditions required for effective integration in Portugal. These insights are potentially transferable to other contexts in countries with the same level of maturity regarding the integration of genomics in nursing. The analysis suggests that nursing’s holistic, person and family-centered approach position the profession to play a pivotal role in the translation of genomic advances into clinical practice. Strengthening genomic literacy, formal recognition of competencies, and structured pathways for education and regulation were identified as essential to ensure safe, equitable, and sustainable implementation. Continued collaboration between professional bodies, educators, and policymakers will be key to advancing genomics-informed nursing practice.

## Supplementary Information

Below is the link to the electronic supplementary material.

Supplementary File 1 (DOCX 17.7 KB)

Supplementary File 2 (DOCX 43.3 KB)

## Data Availability

The data that support the findings of this study are available on request from the corresponding author. The data is not publicly available due to privacy or ethical restrictions.

## Declarations

### Positionality

As researchers, we acknowledge that our social, professional, and cultural positions actively shaped both the data generation process and the interpretative lens through which the analysis was conducted. Our team is composed of women with diverse disciplinary and professional backgrounds, including nursing, psychology, and genetic counselling, spanning different academic levels, from clinicians and educators to senior professors, which brought multiple layers of insight and experience to the study. Our distinct cultural and professional contexts, shaped by our backgrounds in Portugal, Cuba, and the United States, also informed how we understood and engaged with the field of genomic healthcare. Reflecting on our positionality was not an attempt to minimize subjectivity but to acknowledge it as an integral part of meaning-making. We recognize that our perspectives, rooted in nursing, psychology, and genetic counselling, co-constructed the analytic narrative presented in this paper. Through continuous reflexive dialogue, we sought to make these influences explicit and to enhance the transparency and depth of our interpretative engagement.
