# Supplementary material for: A multidisciplinary perspective on advancing genomic nursing in Portugal: roles, barriers and system-level solutions
Source: J Community Genet. 2026 Feb 17;17(2):30. doi: 10.1007/s12687-026-00861-3 (PMC12909635; doi:10.1007/s12687-026-00861-3)
Supplement: Supplementary file 1 — (DOCX 17.7 KB) [file 12687_2026_861_MOESM1_ESM.docx]

**Appendix A. Focus Group Guide**

| **Question** | **Duration** | **Strategy** | **Tool/Link** |
| --- | --- | --- | --- |
| 1. Do you consider it relevant for nurses to have specific knowledge in the field of genomics? Why? | 5’ | Participant comments |  |
| 2. Based on your clinical practice, what genomic knowledge do you consider essential for nurses? | 10, | Response box | Mentimeter |
| 3. What specific competencies do you think nurses should develop to apply genomics in healthcare? | 10’ | Response box | Mentimeter |
| 4. In your opinion, how would you describe nurses’ preparedness to deal with genomics-related issues? | 10’ | Participant comments |  |
| 5. If you could propose changes to nursing curricula, what would you include to better prepare nurses for genomics-informed clinical practice? | 15’ | Participant comments |  |
| 6. In your opinion, what should be the nurse’s role within the multidisciplinary team in applying genomics to healthcare? What practical examples are you aware of? | 10’ | Participant comments |  |
| 7. In your opinion, how can collaboration within the multidisciplinary team be optimized in contexts where genomics is applied? | 10’ | Participant comments |  |
| 8. Is there any other aspect you consider important on this topic that we have not discussed? | 5’ | Participant comments |  |
| **Closing:**   - Thank participants for their time and contribution. - Explain the next steps of the study. - Reiterate commitment to confidentiality of data. - Emphasize the importance of participants’ views for improving nursing education. | (5’) | Presentation |  |
