# Supplementary material for: A multidisciplinary perspective on advancing genomic nursing in Portugal: roles, barriers and system-level solutions
Source: J Community Genet. 2026 Feb 17;17(2):30. doi: 10.1007/s12687-026-00861-3 (PMC12909635; doi:10.1007/s12687-026-00861-3)
Supplement: Supplementary file 2 — (DOCX 43.3 KB) [file 12687_2026_861_MOESM2_ESM.docx]

Appendix A – Reflexive Thematic Analysis Reporting Checklist (adapted from Braun & Clarke, 2024)

| **Advice for aspects of the research report/approach to**  **reporting** | **Guiding notes and further explanation** | **Practices, concepts and terminology to avoid** | **Manuscript** |
| --- | --- | --- | --- |
| **The Introduction**  NB: We prefer *Introduction* over *Literature Review* as a section heading, to capture the broader purpose of this section. | | |  |
| ***Background and rationale*** | | | |
| Provide a robust context and rationale for the proposed research in the *Introduction*. | Can discuss existing research, theory, and the wider context; the researcher is understood as *entering a conversation* with existing scholarship. | Critiquing the methodological limitations of existing research from a (post)positivist/ quantitative standpoint; orienting a literature review to  finding a “gap” that the research fills. | *The introduction offers a broad context on genomic healthcare, situating nursing within the evolution of genomic science and interprofessional practice. It outlines the organizational, educational, and regulatory challenges shaping nurses’ participation in genomics, drawing on international frameworks and the Portuguese healthcare context. The rationale emphasizes the underexplored perspectives of multidisciplinary genomic specialists as key to informing nursing education and practice.* |
| Clearly articulate a research question – one that is methodologically coherent. | Can discuss refining an initially broader research question to a more specific one for the paper. | Formulating research questions as hypotheses or expectations about  what might be “found”. | *The research question aligns with an interpretivist and exploratory orientation. It seeks understanding and meaning-making rather than prediction or measurement.* |
| ***“Owning your perspectives”****^6^* | | | |
| Include information on guiding theoretical assumptions and  other (e.g., explanatory) theory  informing the use of TA. | Guiding (e.g., paradigmatic, ontological and epistemological) and other theory should be  coherent with RTA. | (Post)positivism and (simple) realism. | *The study is grounded in an interpretivist and reflexive paradigm consistent with Reflexive Thematic Analysis (RTA). It assumes that knowledge and meaning are co-constructed through dialogue among participants and researchers.* |
| Report in a way that is consistent with stated theoretical assumptions throughout. | Theoretical coherence is evidenced through the use of language and concepts (e.g., around theme development, research subjectivity, data interpretation), the treatment  of data, and use of quality practices consistent with RTA. | Inadvertently “mashing- up” of RTA and (post)positivism/ realism (e.g., assuming data interpretation can be accurate and reliable) – without a clear rationale. | *The manuscript maintains epistemological consistency, using interpretive language and emphasizing researcher subjectivity, co-construction of meaning, and reflexive interpretation aligned with RTA principles.* |
| Evidence methodological coherence/integrity in both the research and the report.^7^ | Theoretical assumptions, research questions, methods/practices of data generation, RTA, and specific orientation to RTA, purpose of research etc. all “fit together”,  conceptually. | Ontological and epistemological confusion (e.g., claiming constructionism but focusing on lived experience and treating language as a transparent window onto this). | *The study demonstrates full methodological coherence between its interpretivist stance, the exploratory question, and the use of Reflexive Thematic Analysis. The theoretical position, design, and reporting are explicitly integrated and transparent throughout.* |

| Show evidence of reflexive practice. | Can discuss researcher professional or personal positioning and experience in relation to the topic, and/or participant group, and/or their role in shaping the research;  use of reflexive journaling. | Evoking researcher bias (positivist), or even researcher influence, in a way that evokes it as *possible* rather than inevitable. | *We embedded researchers’ positionality throughout the manuscript, and underlined how the professional and personal positioning shaped the research.* |
| --- | --- | --- | --- |
| Write in a methodologically coherent style. | A first-person writing style suits RTA, as it “writes in” the researcher and contributes to situated and reflexive  reporting. | A third person writing style – writing the researcher out of the research. | *The manuscript consistently uses interpretive and reflexive terminology aligned with the epistemological assumptions of RTA. The language emphasizes meaning, interpretation, and shared understanding rather than measurement or verification, ensuring theoretical coherence throughout the text.* |
| **The Methodology**  NB: We prefer the theoretically-embedded term *Methodology* as a section header, over the proceduralist term *Method*. | | | |
| ***Participants/data items*** | | | |
| Describe selection of participants/data items. | Should include criteria for selection and/or recruitment strategies and settings. | Terms  “sample/sampling”, which connote “sampling” from a population (for the purpose  of statistical generalization). | *The selection of the participants and the criteria for inclusion and recruitment are described in the manuscript.* |
| Describe number of participants/data items; provide a rationale or explanation around dataset or participant group size/composition. | Non-positivist qualitative concepts, such as “information power” or sufficiency offer conceptually appropriate justifications for  “dataset” or “participant group” size and  composition.^8^ | Justification based on saturation (simple realist), or statistical models (positivist); reporting rates of non- participation (an indicator of the representativeness of the “sample” in quantitative  research). | *A detailed table with the number and profile of participants is included in the manuscript.* |

| Discuss characteristics of participants/data items. | Balance the need to “situate the participant group” with participant anonymity (e.g., aggregate or report minimal demographics where appropriate).^6^ | Tables with each participant’s demographic information listed line- by-line. | *Participants profile is described, with minimal demographics to assure anonymity.* |
| --- | --- | --- | --- |
| Detail ethical approval and ethical code/principles followed, participant informed consent, etc. | Ethical discussion usually includes institutional ethical approval (if needed), but may include wider principles; providing research materials (participant information, consent form, etc.) in supplementary materials may  be useful to support reflexive openness. | Compromising participant anonymity by the details provided. | *The study was approved by the i3S-Institute for Research and Innovation in Health, University of Porto, Portugal (*Ref. 5/CECRI/2025*).*  *Participant informed consent was obtained.* |
| ***Dataset generation***  NB: We prefer the term *generation* over *collection* to capture the active role of the researcher and that  data don’t pre-exist research *as* data, but *become* data through research practices. | | | |
| Provide some rationale for method(s) for data generation/data item sources chosen. | Discuss why the method(s) of data generation/data source was a good fit with the research question, participant group, guiding theory, etc. If multiple data sources are used, any rationale for combination should be conceptually appropriate (e.g.,  crystallisation^9^). | Triangulation as a rationale for different data sources (realist). | *Online focus groups with multidisciplinary genomic specialists provided rich, dialogic, and contextually grounded data about how genomics is organized and practiced in Portugal. This method aligned with the interpretivist stance and RTA principles by fostering collective reflection, interaction, and co-construction of meaning among professionals from diverse institutional and disciplinary backgrounds.* |
| Describe development and/or characteristics of data generation tool(s). | Include tool(s) in supplementary materials when possible; discuss piloting if used, and any changes following piloting, or during  data generation. | Using an existing tool with the aim of replicating existing “findings”, or developing and describing a tool in a way that is intended to facilitate future replication (positivist). | *Data were generated through semi-structured focus group discussions guided by an interview schedule developed from the study aims and relevant literature. The guide explored service organization and workflows, professional roles and interfaces, competencies for genomic care, gaps in nursing preparation, and strategies for integration. It was circulated in advance to promote reflection and allow participants to prepare examples. The tool was intentionally flexible to support dialogic interaction and depth while maintaining alignment with the research question and RTA principles.* |

| Include details such as modality and/or setting of data generation, time frame, and other pertinent procedural information. | Relevant information includes: the mode of a data generation tool (e.g., video call focus groups; chat- based interviews); the context of data generation (location; timeframe) – where this doesn’t compromise participant anonymity; and mode of recording interactive  data generation. | Standardization as a gold standard (realist); justifying an aimed for standardization in data generation tools as a means to facilitate the  “reliability” or  “accuracy” of the research; treating a lack of standardization in data generation method, modality or setting as a problem, a potential source of “bias”. | *We included in the manuscript details regarding de mode of data generation – online focus group, use of Zoom, and simultaneous transcription.*  *Interview guide presented as Appendix A.* |
| --- | --- | --- | --- |
| Describe who conducted any interactive data generation (which author or research role), and how. | Can include what, if anything, the researcher disclosed about their personal or professional positioning or motivation; what skills and experience they brought; note  researcher’s relationship with participants prior to, during and after the  research. | Seeking standardization (e.g., through the training of researchers) in interactive data collection; treating non- standardisation as a threat to “reliability” or “accuracy”. | *We described who conducted the interactive data generation, detailing the researchers’ positioning and their professional experience.* |
| Describe the size/scope of dataset and dataset items. | Such as the range and average length for interviews/focus groups; range and average word length  for textual data items. | Equating data *quantity*  with data *quality.* | *We described the length of the focus groups.* |

| Describe, and if relevant explain, any preparation of data for analysis. | Such as method of transcription of audio/video data (a transcription key can go in supplementary materials); changes and “corrections” – such as why typographical errors in written data were corrected; system for removing any identifying information; use of pseudonyms and/or data  codes. | Describing transcription as “verbatim” or “orthographic” with no further details; using  edited or “cleaned up” data without acknowledgement of this; participant validation of the  “accuracy” of  transcripts (realist). | *We described that all the focus group were audio recorded and transcribed using Zoom transcription tool.* |
| --- | --- | --- | --- |
| ***Data analysis*** | | | |
| Provide some rationale for use of RTA, and, where relevant, for combining RTA with other approaches and procedures. | Any combining of RTA with other method/ologies or procedures should be warranted, rather than based on a misunderstanding of RTA, and conceptually coherent  (unless clearly justified). | Citing generic characteristics of RTA (e.g., accessible, flexible) without explaining how they were *relevant* to the study; using a codebook without acknowledging this is not part  of RTA and justifying its use. | *RTA was chosen for its methodological coherence with the study’s interpretivist stance and its suitability for exploring complex, practice-based experiences of multidisciplinary genomic specialists. This approach enabled an in-depth, reflexive understanding of shared meanings surrounding nurses’ roles in genomic healthcare.* |
| Describe specific orientation to RTA. | Locate RTA on dimensions of inductive<>deductive and semantic<>latent. | A generic discussion of TA (or even RTA), not specifically situated in relation to the study or  approach. | *We adopted a reflexive, interpretive orientation to RTA, consistent with Braun and Clarke’s approach.* |
| Discuss how the researcher(s) engaged with the analytic process. | Provide a specific and situated account of the analysis process; use supplementary materials to provide a fuller  account of the analytic  process. | Offering a generic description of the six phases of RTA in lieu of an account of analytic process. | *The researchers acknowledged their positionality, identities, and professional trajectories as shaping their perspectives and analysis, consistent with the reflexive nature of RTA.* |
| Where more than one  person is involved, describe who analyzed the data (author or research role). | Role(s) or involvement throughout the process should be discussed; where coding was collaborative, what this involved and how differences in coding and theme development were tackled, should be  included. | Use of inter-coder  agreement measures, consensus coding approach (positivist). | *All transcripts were initially coded independently by two researchers, followed by iterative discussions with a third to refine interpretation and theme development. Differences were addressed through reflexive dialogue rather than consensus coding, consistent with RTA’s interpretive orientation.* |

| Use language to describe the process and products of RTA that is coherent with the values and assumptions of RTA. | Language should convey the *active* role of the researcher(s) in “generating”, “crafting”, “constructing”, “creating”, “producing” or “developing” themes; language around themes should evokes them as *products* of a researcher-data process. | Passive language of discovery, such as “emerging”, “found”, “identified”, “discovered” – these evoke themes as  “diamonds scattered in the sand” (p.  740)^10^; unexplained use of language and concepts from other approaches, such as emergent or superordinate  themes (IPA), or line-by- line and/or open coding and constant comparison (grounded  theory). | *Themes were conceptualized as patterns of shared meaning co-constructed between researchers and participants, not as objective findings. We used language compatible with RTA, not using passive language.* |
| --- | --- | --- | --- |
| **The Analysis**  NB: We prefer the heading *Analysis* over *Findings*/*Results*. *Findings* implies the researcher “found”, “discovered” or “identified” pre-existing themes. *Results* is strongly associated with the outputs of  statistical analysis. | | | |
| ***Reporting the data analysis*** | | | |
| Provide an overview of themes or thematic structure. | Overviews can include a list, map or table of themes to preview the analysis. | An unclear thematic structure, including unexplained headings in the *Analysis*. | *A thematic map illustrating relationships between themes and subthemes is presented in Figure 1. Three overarching themes were constructed, each representing shared meanings across participants’ experiences and perspectives on nursing roles and the integration of genomics into education and practice.* |
| Ensure theme conceptualization is appropriate to RTA, and any divergences are justified and  explained. | In RTA, themes report shared meaning, united around a central organizing concept that differs for each theme. | Topic summaries; data generation questions reported as “themes”. | *Themes were conceptualized as interpretive patterns of shared meaning organized around central concepts, reflecting participants’ collective understanding of nurses’ roles, barriers, and systemic conditions shaping genomic integration.* |

| Name themes appropriately. | Use theme names that capture the “essence”  or “story” of each themes; brief data quotations can be used. | (One-word) theme names that only identify a topic, and offer no story (evoking topic summaries). | *Themes were named to convey the central narrative of each pattern of meaning, capturing the essence of participants’ reflections on nurses’ roles, barriers to integration, and system-level strategies for advancing genomics in nursing.* |
| --- | --- | --- | --- |
| Report themes in sufficient depth and detail. | As RTA is an interpretative method, themes should be multifaceted, and contain both data and analytic narrative; if useful, additional data extracts may be included  in supplementary  materials.^7^ | Thin, one dimensional themes, effectively conflating codes and themes; large number of themes relative to the length of the manuscript. | *Each theme is presented with subthemes and extensive illustrative participant quotes, providing depth and contextual richness* |
| Use subtheme judiciously. | Themes are the main analytic purpose, and should be multifaceted; only use subthemes where doing so highlights an important facet  or aspect of the central  concept of a theme. | Fragmenting the analysis through overuse of subthemes, and an overly  elaborated/“bitty”  thematic structure. | *Subthemes were used where needed to capture more specific dimensions within each overarching theme*. |
| Ensure the analytic narrative explains the meaning and significance of the data. | For RTA, each theme needs an analytic narrative that outlines its meaning and importance in relation to the topic, research question and dataset; the reader needs to be told about why/how data excerpts matter and “evidence” the theme; the A*nalysis*  section also needs to convey the *overall* story of the analysis. | Frequency counts as a justification for themes presented; simple paraphrasing of data as “analytic narrative”; treating data meaning as self- evident (data are assumed to speak for themselves); “arguing” with the data (treating the data as something to [dis]agree with, rather than to interpret and make sense of). | *The analytic narrative interprets how professional, organizational, and educational contexts shape nurses’ engagement with genomics. Each theme is discussed in relation to the research question, highlighting the significance of participants’ perspectives and illustrating how their accounts inform a broader understanding of genomic integration in nursing.* |

| Provide an appropriate *balance* of analytic narrative and data extracts – both data  extracts *and* analytic narrative matter. | The rich descriptive and/or interpretative story of the analysis needs to be woven around sufficient analytic extracts from across the dataset. | Presenting either a long string of data extracts with barely any analytic narrative, or *only* the researcher’s narrative summary of the theme, without any data extracts to support it. | *The analysis integrates researcher interpretation with rich participant quotes, ensuring balance between narrative and evidence.* |
| --- | --- | --- | --- |
| Demonstrate coherence between analytic narrative and  illustrative/evidentiary data extracts. | Data extracts should convincingly and compellingly evidence the analytic claims. | Mismatches between data extracts and analytic claims;  not countering obvious alternative readings of the data | *Quotes are used to illustrate and ground each analytic point, ensuring alignment between interpretation and participant accounts.* |
| Integrate existing research and theory into the analytic narrative. | In RTA, an interpretative analytic narrative is enriched by incorporating relevant existing research and theory into the reporting of themes, reflecting notions of contextualized meaning, and contributing to an ongoing  “conversation” about a topic. | The positivist tradition of separating a description of analytic “Results” and their interpretation with reference to scholarship and theory in a  “Discussion” section. | *The analytic narrative integrates relevant literature and theory to contextualize themes, situating participants’ perspectives within broader discussions on nursing genomics, professional education, and interprofessional collaboration. This integration deepens interpretation and positions the findings within the global discourse on genomics-informed nursing practice.* |
| **The Final Section – A General Discussion or “Conclusions”**  NB: We don’t have a preference for what a final section of an RTA report is called, and it depends on the context and the focus and purpose of the study – the heading *Conclusion* may evoke a certainty that isn’t appropriate; *Implications* may be useful; *Final Considerations* or *Reflections*  may work, as might *General Discussion*. | | | |
| ***Quality, evaluation and conclusions*** | | | |
| Draw analytic conclusions across themes. | Orient to the “so what” of the *overall* analysis – the “point” of the story told; this might include discussion of implications for practice and  “actionable” outcomes.^11^ | Repetitive theme-by- theme integration of the analysis with existing literature; no overall conclusions drawn; no overall analytic story. | *The final discussion synthesizes insights across the three themes, highlighting how educational, regulatory, and organizational factors intersect to shape nurses’ participation in genomic healthcare. It emphasizes actionable implications for strengthening genomic literacy, formal recognition, and interprofessional collaboration within nursing practice.* |

| Discuss implications or directions for future research. | Any suggestions for future research should stem from the analysis and be evidence- based (e.g., provide grounds for other groups potentially having different experiences or views) rather than generic. | Generic recommendations for other research, such as with a different “population”. | *The manuscript discusses implications grounded in the analysis, emphasizing the need for educational reform, regulatory recognition, and system-level alignment to enable genomic integration in nursing. It suggests future research involving broader nursing and policy perspectives to further explore structural conditions for genomics-informed care.* |
| --- | --- | --- | --- |
| Use and report quality practices coherent with RTA. | Ensure evaluation of research quality deploys conceptually coherent notions, such as: member reflections; crystallisation;^9^ others serving as a critical friend/sounding  board to enhance insight;^12^ reflexive journaling. | Incoherent quality measures such as: member checking/participant validation; triangulation (realist); the use of theme agreement/consensus among researchers or corroboration of themes by another  researcher (positivist). | *We used positionality and reflexivity and followed Braun and Clarke’s six- step framework to ensure quality and rigor.* |
| Evaluate the research from a *Big Q* standpoint. | Such evaluation might including considering how the *specifics* of the study may have shaped the research produced (for example, the characteristics and context of the participant group/dataset; the methods and modalities  for generating the data); situatedness should not be treated as a limitation. | Evaluations and descriptions of limitations that orient to quantitative or positivist norms, such as reference to lack of generalisability – positioned as a limitation, and equated only with statistical generalisability^13^ – or a “small” (by implication non-ideal) and “unrepresentative” “sample”. | The study adopts an interpretivist, reflexive epistemology. |
| Include reflections on research process and practices, including researcher  reflexivity. | Some consideration of the researcher(s)’s role in shaping  the research and the knowledge generated is an important quality marker. | Reference to researcher bias/influence (positivist). | The researchers explicitly acknowledge their positionality, identities, and trajectories as part of the analysis |
